# An Electrochemical Sandwich Immunosensor Based on Signal Amplification Technique for the Determination of Alpha-Fetoprotein

**DOI:** 10.3389/fchem.2020.589560

**Published:** 2020-09-16

**Authors:** Changming Shen, Lin Wang, Hongyan Zhang, Shaojuan Liu, Jianwei Jiang

**Affiliations:** ^1^Cancer Hospital of the University of Chinese Academy of Sciences (Zhejiang Cancer Hospital), Hangzhou, China; ^2^Institute of Cancer and Basic Medicine (IBMC), Chinese Academy of Sciences, Hangzhou, China; ^3^The Third Affiliated Hospital of Zhejiang Chinese Medical University, Hangzhou, China; ^4^The Third People's Hospital of Hangzhou, Hangzhou, China

**Keywords:** sandwich immunosensor, alpha-fetoprotein, electrochemical analysis, cancer biomarker, glassy carbon electrode

## Abstract

The synthesis of Au nanocubes is used to label alpha-fetoprotein antibody (anti-AFP) and horseradish peroxidase (HRP) to form an immune complex for antibody detection. Graphene oxide-methylene blue-gold nanoparticles (GO-MB-AuNPs) nanocomposites were used as the immunosensing platform. This proposed sandwich-type immunoassay shows good performance. This method establishes a feasible amperometric immunoassay method for sensitive analysis of AFP in serum samples. Under the optimal experimental conditions, the DPV current response of the immunosensor is proportional to the logarithmic value of the AFP concentration. The linear detection range can achieve to 0.005–20 ng/mL with a detection limit of 1.5 pg/mL. The proposed immunosensor has good precision, selectivity and stability, and can be used for AFP determination in clinical tests.

## Introduction

Alpha-fetoprotein (AFP) is a glycoprotein, mainly produced by embryonic liver cells. About 2 weeks after birth, AFP disappears from the blood. Under normal circumstances, the content of AFP in adult serum is <25 ng/mL. However, it is elevated in the blood of about 80% of liver cancer patients. When liver cells become cancerous, they restore their ability to produce AFP. When the condition gradually worsens, the content will increase substantially (Li et al., 2017, 2020; Yang et al., 2018; Ma L. et al., 2019). Therefore, AFP can be used as a specific clinical detection index for the diagnosis of primary liver cancer. AFP can be detected in multiple channels, generally including fluorescence analysis, enzyme-linked immunoassay, chemiluminescence analysis, chromatography, radioimmunoassay, enzyme-labeled electrophoresis, and electrochemical analysis. From the analysis of markers, it can be divided into labeled AFP sensors and label-free AFP sensors (Fang et al., 2017; Wang et al., 2017; Liu et al., 2018; Fu et al., 2019a; Shamsadin-Azad et al., 2019).

Immunosensor is a combination of sensing technology and specific immune response to detect the reaction between antigen and antibody. A healthy body can defend against the invasion of microorganisms, viruses, and other harmful substances through a variety of mechanisms, including natural immunity and acquired immunity. Antigens are exogenous substances that can induce specific immunity, which can stimulate the immune system of animals and plants and produce an immune response dominated by antibodies and lymphocytes (Alizadeh et al., 2018; Karimi-Maleh et al., 2020a,b). Immunosensor is a detection device designed using the principle of specific recognition and binding of antigen and antibody. Antigen and antibody molecules are fixed on the surface of the electrode in some form, and form a stable complex with the corresponding antibody or antigen to be detected (Wang et al., 2018; Fu et al., 2020a,b; Karimi-Maleh and Arotiba, 2020). These compounds can cause changes in sensor electrical signals, such as electrode potential or current and capacitance.

Immunolabeling mainly refers to labeling antibodies or antigens in specific reactions to catalyze reactions and improve sensor sensitivity. Common markers include luciferin, enzymes, radioisotopes, nanomaterials, and electronic dense substances. The label sensor is highly sensitive and specific, which has been widely used (Peng et al., 2018; Fu et al., 2019b; Ge et al., 2019; Ma N. et al., 2019; Hu et al., 2020; Ying et al., 2020). When technology enters the nano-era, a large number of nanomaterials were used in biosensors (Baghayeri et al., 2018; Feng et al., 2020; Hojjati-Najafabadi et al., 2020; Hou et al., 2020; Karimi-Maleh et al., 2020c). Therefore, in addition to the above immunolabels, many researchers use nanomaterials as labels to expand the antigen and antibody immune response signals.

In this work, we synthesized Au nanocubes, which are used to effectively label biomacromolecules in electrochemical immunosensors and immunoassays. At the same time, they can also have a significant catalytic effect on the response of the sensor, thereby significantly improving the electrochemical response of the sensor. We then use Au nanocubes to immobilize both AFP antibody and horseradish peroxidase (HRP) as the detection antibody labeling material. In the presence of AFP antigen, a sandwich immunosensor analysis mode is used to perform sensitive and quantitative detection of AFP. The immunosensor has few consumables, high sensitivity and high selectivity, and can be applied to clinical detection.

## Materials and Methods

Graphene oxide (GO) was purchased from Xianfeng Nano Co., Ltd. HAuCl_4_·4H_2_O was purchased from Shanghai Yuanye Biological Co., Ltd. Methylene blue (MB), cetyltrimethylammonium bromide (CTAB), NaBH_4_, and trisodium citrate were purchased from Sinopharm Chemical Co., Ltd. The AFP standard solution and mouse monoclonal antibody (anti-AFP) were purchased from Zhengzhou Bosai Biotechnology Co., Ltd. All reagents were analytical grade.

The electrochemical experiment was carried out on the CHI 760C electrochemical analyzer (Shanghai Chenhua Instrument Co., Ltd., China). Three electrode system was adopted: modified electrode as working electrode, platinum wire electrode as counter electrode, saturated calomel electrode as reference electrode (SCE). The solution used in the electrochemical impedance analysis was 0.1 M KCl containing 10 mM K_4_[Fe(CN)_6_] and 10 mm K_3_[Fe(CN)_6_]. Both cyclic voltammetry (CV) and differential pulse voltammetry (DPV) experiments use 0.1 M PBS as the test solution (containing 0.1 M KCl, pH 6.4).

The preparation of Au cubes was according to the literature. The formed Au cubes were then dispersed into 0.1 M PBS with the addition of HRP and anti-AFP. The mixed solution was stirred under 4°C overnight to form AuC-HRP-anti-AFP.

GO-MB-AuNPs were prepared by mixing of three substances into a 0.1 M PBS overnight. The composite was obtained using a centrifugation process. Then, a certain amount of GO-MB-AuNPs was dip coated on the GCE surface and dried naturally. Ten microliter of anti-AFP was dip coated on the modified electrode and incubated overnight. Then, the electrode was immersed into a BSA solution for removing the excess of anti-AFP. During the AFP sensing, the standard AFP solution was dip-coated on the above-mentioned electrode and incubated for 30 min. Then, the AuC-HRP-anti-AFP was dip-coated on the electrode to forming the sandwich sensor. The DPV scan was recorded in a 0.1 M PBS (containing 4 mM H_2_O_2_).

## Results and Discussion

**Figure 1:**
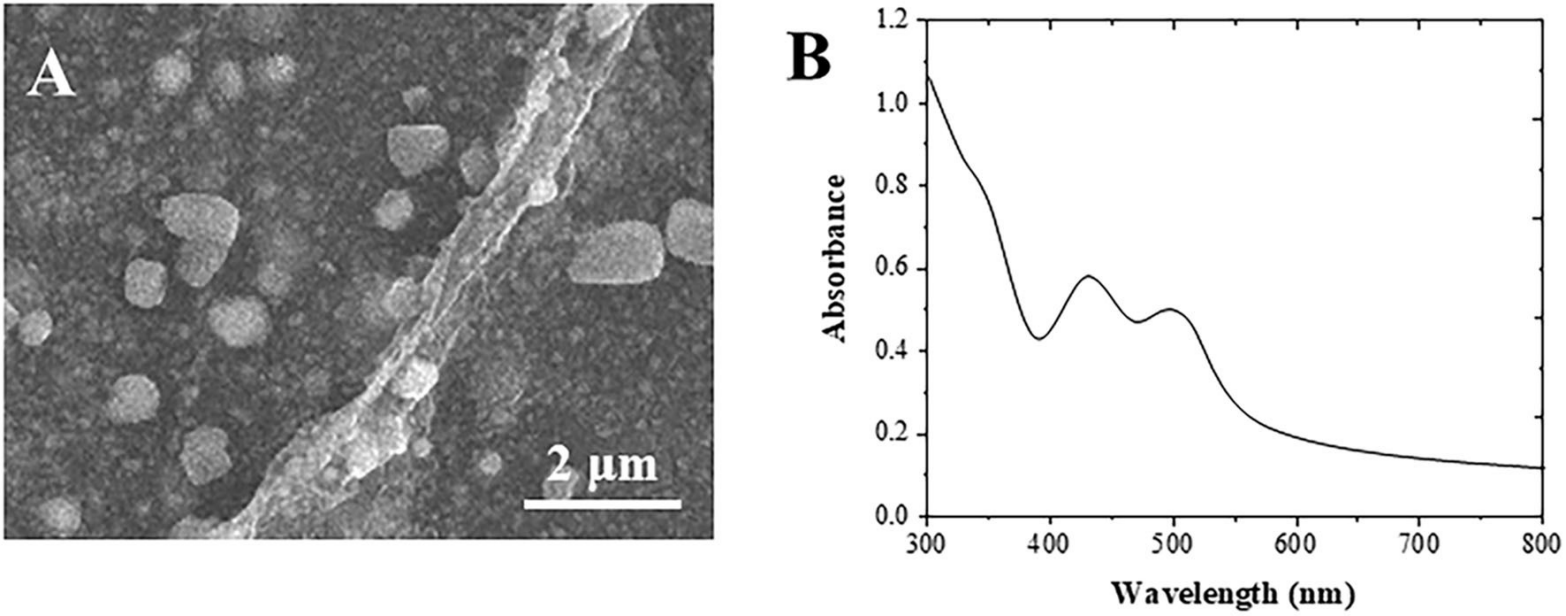
**(A)** SEM and **(B)** UV-vis spectrum of Au cubes.

Figure 1A shows the SEM image of the formed Au cubes. The diameter of the Au cubes was at an average of 80 nm, in which the structure facilitates the immobilization of anti-AFP antibody and HRP enzyme (Yu et al., 2017). Different substances can produce different UV-visible absorption peaks due to their different structures. It can be seen from Figure 1B that there are two obvious absorption peaks at 460 nm and 532 nm in the UV absorption peak of Au cubes (Zhang et al., 2020).

**Figure 2:**
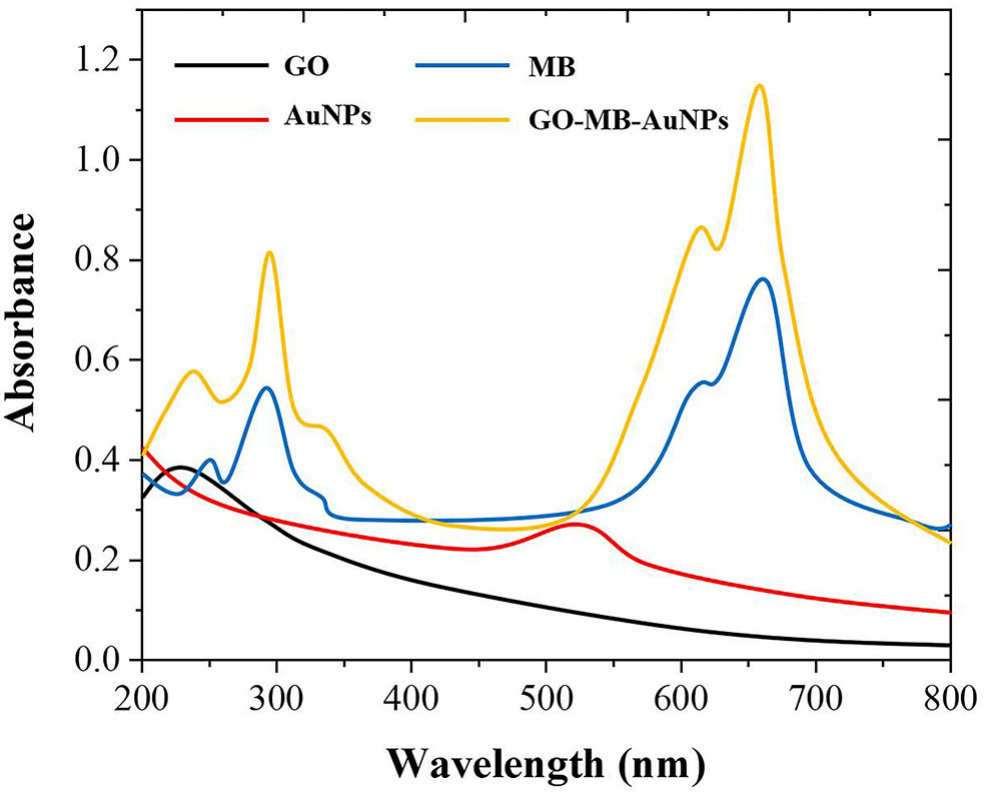
UV-vis spectra of GO, MB AuNPs, and GO-MB-AuNPs nanocomposite.

As shown in Figure 2, GO has an adsorption peak around 230 nm, while MB has two absorption peaks at around 290 and 650 nm. AuNPs have specific ultraviolet absorption peaks between 500 and 600 nm due to their different particle sizes. The preparation of GO-MB-AuNPs nanocomposites was investigated by UV-vis spectrometer as well (Yang et al., 2017). There are four absorption peaks of GO-MB-AuNPs nanocomposites, which are located at 232, 290,527, and 644 nm respectively, indicating the formation of GO-MB-AuNPs nanocomposites.

**Figure 3:**
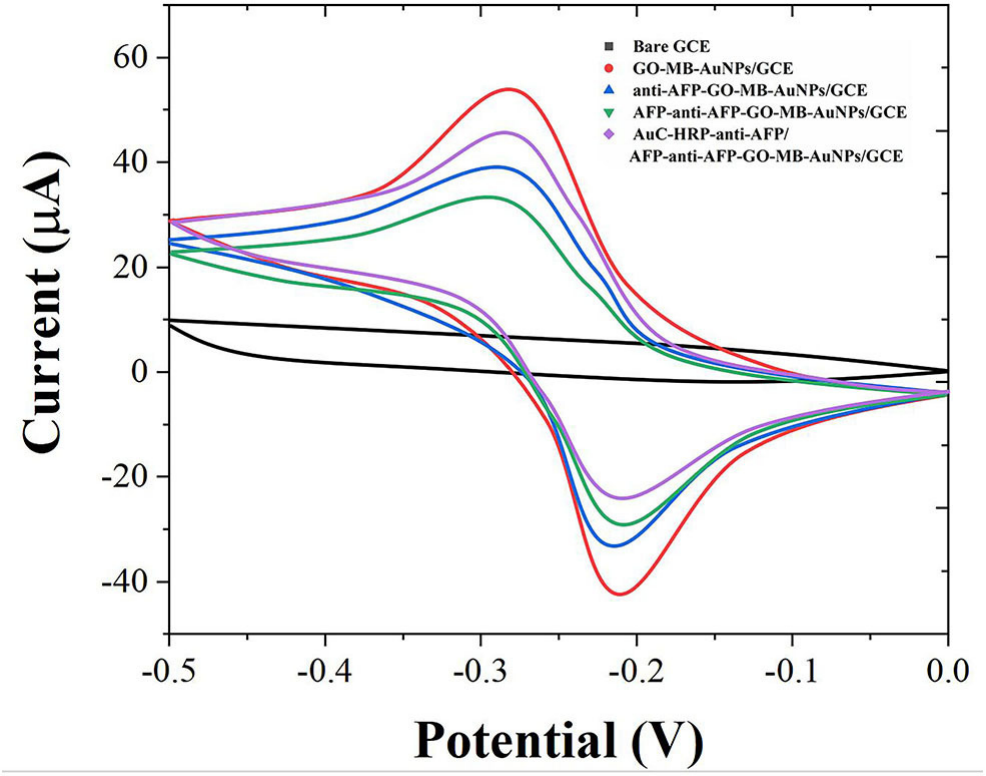
CVs of bare GCE, GO-MB-AuNPs/GCE, anti-AFP-GO-MB-AuNPs/GCE, AFP-anti-AFP-O-MB-AuNPs/GCE, and AuC-HRP-anti-AFP/AFP-anti-AFP-O-MB-AuNPs/GCE.

Cyclic voltammetry (CV) was used to investigate the electrochemical behavior of modified electrodes at different preparation stages in pH 7.0 PBS (containing 4 mM H_2_O_2_). As shown in Figure 3, the bare GCE has no redox peak in the PBS solution. When the GO-MB-AuNPs nanocomposite material was modified on the electrode surface, a pair of stable redox peaks appeared on the electrode (Zhang et al., 2019). This is the redox of MB immobilized on graphene oxide. After GO-MB-AuNPs capture the anti-AFP antibody and AFP antigen, the redox peak current on the electrode decreases successively, which proves that the antibody and the generated antigen-antibody immune complex have poor conductivity and hinder the electrons transfer on the electrode surface.

**Figure 4:**
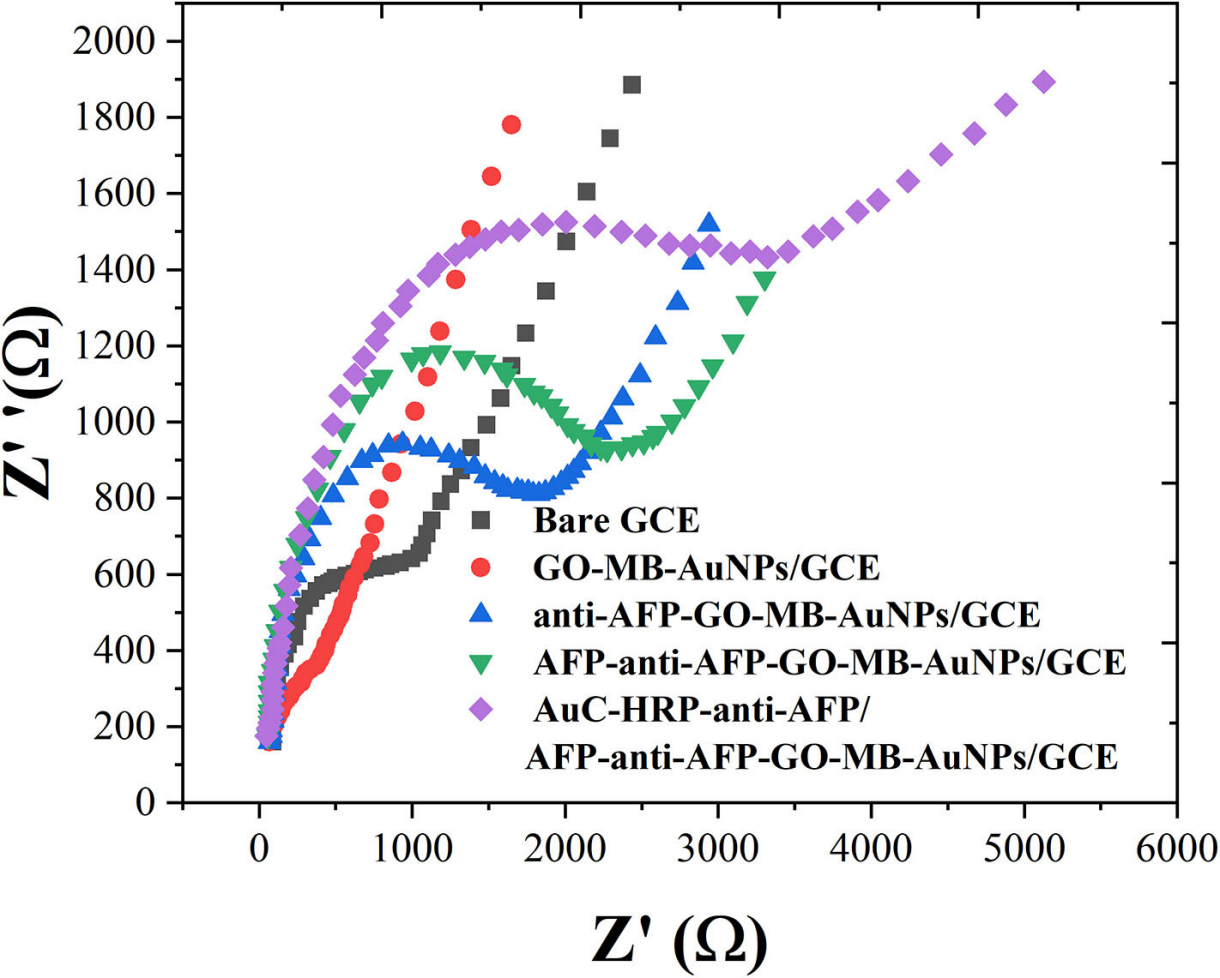
EIS of bare GCE, GO-MB-AuNPs/GCE, anti-AFP-O-MB-AuNPs/GCE, AFP-anti-AFP-O-MB-AuNPs/GCE, and AuC-HRP-anti-AFP/AFP-anti-AFP-O-MB-AuNPs/GCE.

EIS can give information about the impedance change on the interface of the electrode during the modification process (Naderi Asrami et al., 2020). The semicircle diameter of EIS is equal to the electron transfer resistance (R_et_), which controls the electron transfer kinetics of the redox probe on the electrode surface (Campuzano et al., 2017; Ganbat et al., 2020). The EIS results were consistent with the CV results, as shown in Figure 4. These results fully indicate that the electrode preparation was successful and the sandwich-type immunosensor can be formed in turn. The synthesized Au nanocubes have high conductivity and electron transfer efficiency, which facilitates the exchange of electrons between the solution and the substrate electrode.

We used the CV method to optimize the amount of modifier, incubation time, pH, and H_2_O_2_ concentration of the GO-MB-AuNPs nanocomposite. The experimental results show that as the amount of GO-MBAuNPs nanocomposite gradually increases, the peak current gradually increases and the peak current reaches the maximum at 5 μL. When it continues to increase, the peak current decreases instead. Therefore, 5 μL is the best amount of modifier in this work. The incubation time is the time for the antigen-antibody immune reaction on the electrode surface. The results show that the maximum current can be reached when the antigen and antibody reacted for 30 min. There is almost no change in peak current over 30 min. Therefore, we selected 30 min as the incubation time. At the same time, we tested the peak current response of the electrolyte immunosensor at different pH. At pH 5.5–7.0, the peak current of the sensor gradually increases and then gradually decreases. The results prove that pH 7.0 is the optimal pH for the antigen-antibody reaction in this environment, which can ensure the good activity of antigen and antibody. HRP catalyzes H_2_O_2_ as an important part of signal amplification. The peak current of the sensor increases as the concentration of H_2_O_2_ increases, and reaches the maximum value at 4 mM. Therefore, 4 mM is used as the optimal concentration of H_2_O_2_ in subsequent experiments.

**Figure 5:**
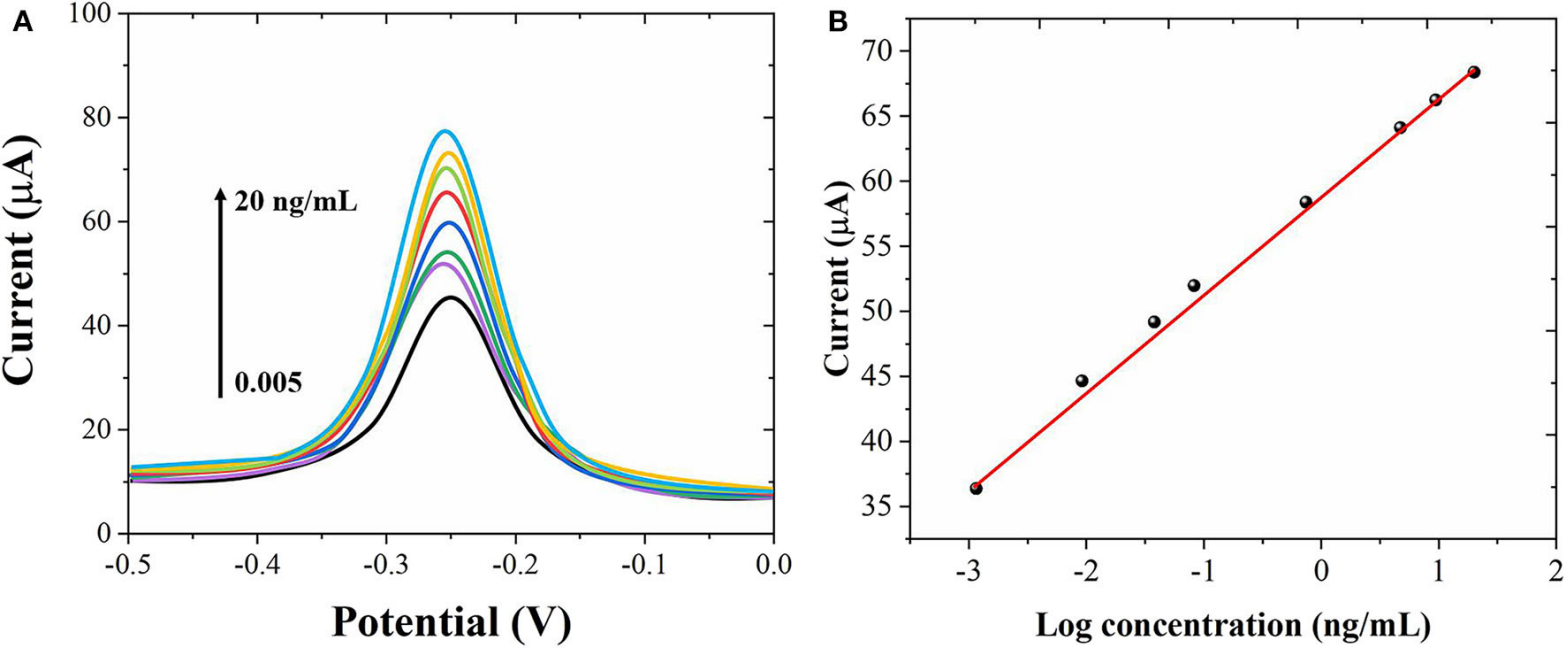
**(A)** DPV curves of the proposed immunosensor for different concentrations of AFP detection from 0.005 to 20 ng/mL. **(B)** Corresponding linear relationship between the AFP concentrations and peak currents.

Under the optimum conditions, we used the proposed immunosensor to detect AFP standard solutions of different concentrations. Figure 5A shows the DPV graph of the sensor to different concentrations of AFP. When the concentration of AFP is between 0.005 and 20 ng/mL, the peak current of DPV increases with the increase of AFP concentration with a good linear relationship. Figure 5B shows the linear relationship between the AFP concentrations and the peak current. The limit of detection was calculated to be 1.5 pg/mL. Compared with other related reports (Jiao et al., 2016; Wei et al., 2016; Yuan et al., 2017; Li et al., 2018), this experimental result has a wider linear range and lower detection. The results show that Au nanostructures can be used as a labeling material to immobilize more antibodies and enzymes, which greatly improves the sensitivity and signal response of the electrode, so that the electrode has a wider linear range and lower detection limit.

The proposed electrochemical immunosensor was used to detect the AFP in the serum samples by the standard addition method. The results were shown in Table 1. The recovery rate obtained from the experimental results was 97.83–101.75%, indicating that the immunosensor has the potential for clinic AFP determination.

**Table 1:** Detection of AFP in serum samples with recovery rate.

**Concentration (ng/mL)**	**Add (ng/mL)**	**Found (ng/mL)**	**Recover (%)**	**RSD (%)**
2	2	4.07	101.75	3.37
5	2	6.87	98.14	5.20
10	2	11.74	97.83	2.66

The reproducibility is a key issue of the electrochemical sensor. For this purpose, the current response of the four sensors prepared at the same conditions was measured. The results displayed that the relative standard deviation (RSD) of electrochemical response for four sensors was about 3.31%, indicating that the immunosensor had a good reproducibility. Meanwhile, the RSD obtained from six successive measurements for each electrode was ~2.61%, suggesting acceptable repeatability.

## Conclusion

Experimental results show that Au cubes were ideal material for immobilizing antigen-antibody, which can greatly improve the response signal of the electrode. The proposed sandwich-type immunosensor has higher sensitivity. The detection of AFP also has a wide linear range and a low detection limit, and a good recovery rate is obtained when the actual sample is detected.

## Data Availability Statement

The original contributions presented in the study are included in the article/supplementary material, further inquiries can be directed to the corresponding author/s.

## Author Contributions

JJ contributed the conception and design of the study. CS and LW conducted the electrochemical experiments. CS and HZ performed the statistical analysis. SL and JJ wrote the manuscript. All authors contributed to manuscript revision, read, and approved the submitted version.

## Conflict of Interest

The authors declare that the research was conducted in the absence of any commercial or financial relationships that could be construed as a potential conflict of interest.
